# CAREGIVER RESPONSES TO REMOTE ACTIVITY MONITOR ALERTS OF PERSONS WITH DEMENTIA

**DOI:** 10.1093/geroni/igz038.1192

**Published:** 2019-11-08

**Authors:** Rachel Zmora, Jessica M Finlay, Lauren L Mitchell, Joseph E Gaugler

**Affiliations:** 1 University of Minnesota, Minneapolis, Minnesota, United States; 2 Social Environment and Health, Institute for Social Research, University of Michigan, Ann Arbor, Michigan, United States; 3 Minneapolis VA Health Care System, Minneapolis, Minnesota, United States; 4 University of Minnesota - School of Public Health, Division of Health Policy and Management, Minneapolis, Minnesota, United States

## Abstract

The benefits of technology to alert family caregivers to the needs of persons with Alzheimer’s disease or related dementias (ADRD) are unclear. Previous research indicates that remote activity monitoring (RAM) system alerts can be alternately reassuring and highly stressful for caregivers. We conducted a parallel convergent mixed-methods analysis of 62 primary caregivers of persons with ADRD to evaluate the association between the number of alerts and caregiver outcomes after 6 months. We assessed caregiver-reported usability of the system as well as self-efficacy, sense of competence, and distress as primary outcomes. Linear regression models tested the association between the number of alerts and caregiver-reported usability and primary outcomes. The number of alerts declined over the first 6 months of system use and was not associated with a change in system usability or primary outcomes. Thematic analysis of caregiver-reported perceptions of RAM use simultaneously probed for more in-depth understanding of caregiver experiences of and feelings towards RAM. Preliminary analyses reveal that 28% of caregivers comments were positive, noting benefits such as early warning of health concerns and peace of mind. 34% of comments were neutral or mixed, and 38% were negative. Concerns included false alarms and accidental triggers, losing sleep due to alarms, and difficulties using the system. These findings help characterize the adjustment period to use RAM technology. The mixed-method results inform future research studies and applications of RAM systems so that researchers and caregivers can better understand the initial adjustment period, address concerns, and avoid discontinuing RAM use prematurely.

